# Commentary: Tolerance and Resistance of *Pseudomonas aeruginosa* Biofilms to Antimicrobial Agents-How *P. aeruginosa* Can Escape Antibiotics

**DOI:** 10.3389/fmicb.2019.02164

**Published:** 2019-09-18

**Authors:** Anaïs Soares, François Caron, Manuel Etienne

**Affiliations:** ^1^GRAM 2.0, EA 2656, Normandie Univ, UNIROUEN, Rouen, France; ^2^Microbiology Department, Rouen University Hospital, Rouen, France; ^3^Infectious Diseases Department, Rouen University Hospital, Rouen, France

**Keywords:** *Pseudomonas aeruginosa*, biofilm, antibiotic failure, tolerance, resistance, phenotypic diversity, small colony variants

In a recent article published in Frontiers in Microbiology, Ciofu and Tolker-Nielsen (2019), offered a comprehensive and didactic review on how *Pseudomonas aeruginosa* escapes antibiotic therapy in biofilms through tolerance and resistance mechanisms. Though their paper provided a wide panorama of the current knowledge in this field, we assume that the role of small colony variants (SCV) regarding antibiotic failure could have been discussed.

SCV are a phenotypic subset of the bacterial population surviving in biofilms. They have been described in chronic, persistent and recurrent biofilm-based infections, particularly in cystic fibrosis but also in chronic wound or device-related infections (Häussler et al., 2003; Proctor et al., 2006; Tielen et al., 2014; Johns et al., 2015). They were particularly well-described in *Staphylococcus aureus* (Proctor et al., 1995, 2006; von Eiff et al., 2006; Masoud-Landgraf et al., 2016), but were also identified in other species such as *Pseudomonas aeruginosa* (Häussler, 2004). *P. aeruginosa* SCV have a high biofilm-forming capacity and reduced swimming, swarming and twitching motilities (Déziel et al., 2001). They also have an increased expression of the *psl* and *pel* loci, encoding for Psl and Pel exopolysaccharides, that together with extracellular DNA form the biofilm matrix (Kirisits et al., 2005). Other studies reported that SCV emerged in biofilm cultures before any antibiotic exposure, and were not the sole bacterial population surviving antibiotic treatment (Déziel et al., 2001; Drenkard and Ausubel, 2002; Gloag et al., 2019). Because SCV constitute an adaptation of the parental strain to biofilm growth, contribute to biofilm production, and are encountered in situations where *P. aeruginosa* escapes antibiotics, their role in antibiotic failure through resistance mechanisms, tolerance mechanisms, or both can be questioned.

Recently, Balaban et al. (2019) recalled the definitions of antibiotic resistance and antibiotic tolerance in a consensus statement. Resistance is the ability of bacteria to replicate in the presence of antibiotic, whereas tolerance is the capability of a population to survive exposure to a bactericidal antibiotic without an increase in the MIC. The combination of both mechanisms in biofilms, has been named “recalcitrance” by Lebeaux et al. (2014).

In specific situations, the biofilm growth might favor the selection of resistant mutants. As a fact, the exopolysaccharide matrix might reduce the diffusion in the deepest layers of the biofilm of some antibiotics such as aminoglycosides (Tseng et al., 2013). Bacterial cells entrapped into the matrix might hence be exposed to sub-inhibitory antibiotic concentrations, and resistant mutants might emerge (Kohanski et al., 2010; Jørgensen et al., 2013). In a study by Drenkard and Ausubel (2002), *P. aeruginosa* SCV with higher MICs toward aminoglycosides were recovered in sputum samples of cystic fibrosis patients treated with aminoglycoside-based antibiotic regimen. The mechanism of such increase in the MIC for SCV was not elucidated. As compared to the parent strain, the reduced susceptibility of SCV to aminoglycosides might result from increased interactions between positively charged aminoglycosides and negatively charged matrix components such as extracellular DNA (Chiang et al., 2013). But in this study as well as in others, the MICs increased only for aminoglycosides and not for fluoroquinolones or β-lactams (Singh et al., 2009; Wei et al., 2011).

However, the environmental conditions associated with biofilm growth (oxygen and nutrients limitations) mostly favor the expression of tolerance mechanisms (Walters et al., 2003; Borriello et al., 2004). Among the tolerant cells encountered in biofilms, persister cells are of particular interest (Lewis, 2010; Fauvart et al., 2011; Lebeaux et al., 2014). In various time-kill assays in *P. aeruginosa* biofilm models, a biphasic killing curve, the hallmark of persistence, was observed after antibiotic exposure (Mulcahy et al., 2010; Benthall et al., 2015; Rojo-Molinero et al., 2016). Despite prolonged exposure at high antibiotic doses, viable, and culturable cells were still present in the biofilm while no resistant mutant was detected. Using high doses of antibiotic known to diffuse in the depth of the biofilm, such as ciprofloxacin (Walters et al., 2003; Rodríguez-Martínez et al., 2007), it is likely that antibiotic concentrations rapidly exceeded the mutation prevention concentration, so that pre-existing spontaneous resistant mutants might be eradicated. Regarding tolerance, the role of SCV in biofilm has not been specifically studied even for *S. aureus*. Singh et al. (2009), in a membrane-supported biofilm model, observed the emergence of *S. aureus* SCV among persister cells after treatment with ciprofloxacin, but not after treatment with amikacin, cefotaxime, oxacillin, or vancomycin. To what extent SCV contribute to biofilm persistence remains unelucidated.

From this analysis of the literature, we assume, in accordance with Ciofu and Tolker-Nielsen (2019) that resistance and tolerance mechanisms can both be expressed in biofilm infections. Those two ways for *P. aeruginosa* to escape antibiotics depend on different triggers. Sub-inhibitory antibiotic concentrations and active cell division favor the selection of genetically divergent resistant mutants. Conversely, lethal antibiotic concentrations trigger tolerance mechanisms, through a phenotypic adaptation, toward what could be called the “last hope bacterial response for survival” (Fajardo and Martínez, 2008; Andersson and Hughes, 2014). This phenotypic adaptation combines stress response activation depending on starvation strategies such as stringent response, SOS response or other metabolic pathways (Harms et al., 2016). SCV are part of these antibiotic-recalcitrant biofilm and for sure have specific metabolic characteristics, the increased exopolysaccharide matrix production being at the forefront (Harmsen et al., 2010; Moradali et al., 2017). Whether SCV behave differently than their parental strain regarding resistant mutants or persister cells emergence can to date not be ensured and needs further investigation. However, SCV might play a key role in the expression of persistence mechanisms, through their part in the biofilm structure, and may largely contribute to the environmental conditions associated with antibiotic recalcitrance.

In a daily practice, *P. aeruginosa* SCV can be detected on plate cultures. In most cases, they attest the presence of biofilm-related infection. SCV testing with usual antimicrobial susceptibility testing methods is also feasible, but little difference is expected regarding antimicrobial susceptibility between SCV and other bacterial populations entrapped in the biofilm. Nevertheless, informing clinicians of the presence of SCV seems crucial, as a signal that conditions are gathered for *P. aeruginosa* to escape antibiotics.

## Author Contributions

FC suggested the general commentary and reviewed the manuscript. AS and ME reviewed the literature and wrote the manuscript. All authors listed approved the work for publication.

### Conflict of Interest Statement

The authors declare that the research was conducted in the absence of any commercial or financial relationships that could be construed as a potential conflict of interest.
